# La couverture des pertes de substances cutanées du tiers inférieur de la jambe: à propos de 09 cas

**DOI:** 10.11604/pamj.2019.33.243.18370

**Published:** 2019-07-23

**Authors:** Achraf Bensassi, Redouane Elghadraoui, Anass Zahraoui, Mohammed Elidrissi, Abdelhalim Elibrahimi, Abdelmajid Elmrini

**Affiliations:** 1 Service de Chirurgie Ostéo-articulaire B4, CHU Hassan II, Université Sidi Mohammed Ben Abdellah, 3000, Fèz, Maroc

**Keywords:** Lambeaux musculaires locaux, lambeaux fascio-cutanés, lambeaux en hélice, lambeaux libres, pertes de substances cutanées, tiers inférieur de la jambe, Local muscle flaps, fasciocutaneous flaps, propeller flaps, free flaps, loss of skin substances, lower third of the leg

## Abstract

La couverture des pertes de substances cutanées du tiers inférieur de la jambe est compliquée, et ce dû à la pauvreté des tissus mous adjacents, la précarité de la vascularisation locale et l'exposition osseuse. Nous avons mené une étude rétrospective d'une série de 9 cas de couvertures cutanées du tiers distal de la jambe traitée au CHU Hassan 2 de Fès de l'année 2016 à 2018. L'objectif de notre étude est de relever la particularité des pertes de substances cutanées du tiers inférieur de la jambe, tout en soulignant la difficulté de couverture.

## Abstract (en)

Coverage of loss of skin substances on the lower third of the leg is a challenging problem. This is due to adjacent soft tissues impairment, shortage of local vascularization and bone exposure. We conducted a retrospective study of a series of 9 cases of skin coverage of the lower third of the leg treated at the University Hospital Hassan II of Fez from 2016 to 2018. This study aims to highlight the characteristic of the loss of skin substances on the lower third of the leg, while emphasizing the difficulty of management.

## Introduction

La couverture des pertes de substances cutanées du tiers inférieur de la jambe est compliquée et ce dû à la pauvreté des tissus mous adjacents, la précarité de la vascularisation locale et l'exposition osseuse. Elle résulte le plus souvent d'un traumatisme à haute énergie et plus fréquemment l'apanage du sujet jeune. Le choix du lambeau de couverture dépendra de l'étiologie de la perte, de son siège et de l'état de la zone à recouvrir. Le traitement répondra au principe: parer, fixer, couvrir. L'évolution est favorable, jugée satisfaisante aussi bien sur le plan esthétique que fonctionnel.

## Méthodes

Entre janvier 2016 et décembre 2018, une étude rétrospective a été menée, concernant 09 cas de couverture de perte de substance du tiers inférieur de la jambe, colligé au service de chirurgie ostéo-articulaire B4 de Fès, Maroc. Une fiche d'exploitation a été établie pour chaque patient permettant de faciliter le recueil et l'analyse des différents paramètres épidémiologiques; cliniques, radiologiques, thérapeutiques et évolutifs.

## Résultats

Au terme des résultats nous avons trouvé que les pertes de substances cutanées du tiers inférieur de la jambe sont l'apanage de l'adulte jeune avec une moyenne d'âge au moment du traumatisme de 29 ans et des extrêmes entre 15 et 55 ans. Quatre-vingt-neuf pourcent des cas de notre série étaient de sexe masculin. La cause post-traumatique est la plus retrouvée, s'agissant dans 77,77% des cas d'accidents de la voie publique. Le siège de la perte de substance cutanée était variable avec une prédominance au niveau de la face médiale et au niveau de la face antéro-interne du 1/3 inférieur de la jambe. La surface de la perte de substance cutanée variait entre 40 et 110 cm^2^ en moyenne. Le délai entre la lésion initiale et la réalisation du lambeau est extrêmement variable, il va de 1 à 8 semaines. Le traitement chez nos patients répond au principe: parer, fixer, couvrir. La couverture se fait par différents types de lambeaux, citons [1]: le lambeau neuro-cutané suralse composant d'une palette fascio-cutanée prélevée à la face postérieure du mollet sur l'axe du nerf sural, il comprend les éléments suivants: 1) une palette fascio-cutanée: peau, tissu cellulaire sous-cutané,pédicule neuro-vasculaire et ses ramifications cutanées, fascia; 2) un pédicule: nerf sural et son plexus vasculaire, accompagné de la veine petite saphène, noyés dans le tissu fascio-graisseux sous-cutané.

**Figure 1:**
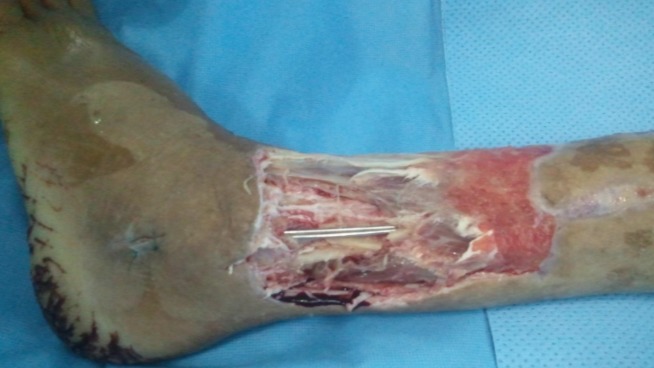
la perte de substance cutanée au niveau de la face antéro-externe du 1/3 inferieur de la jambe après parage

**Figure 2:**
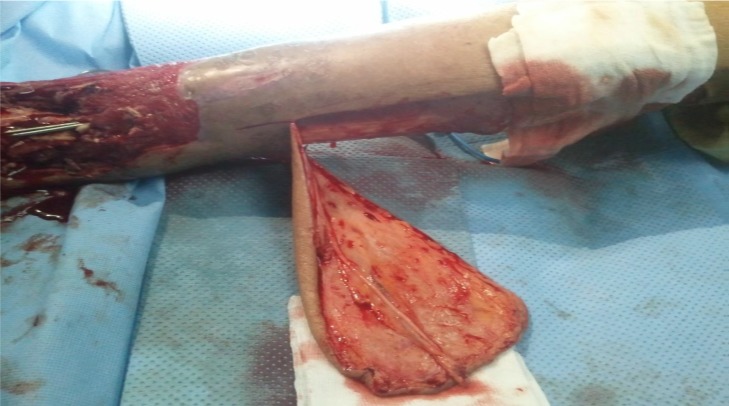
la levée du lambeau fascio-cutané

Le lambeau suscité est classé grade I selon la classification d'Oberlin [2]. Vu sa dissection qui se fait à distance d'un pédicule vasculaire et la possibilité de la réalisation d’une dissection cadavérique préalable. L'installation du patient se fait en décubitus latéral ou dorsal. Un garrot est installé à la racine du membre de manière à dégager le tiers distal de cuisse dans l'hypothèse d'une greffe cutanée. Le décubitus ventral est plutôt réservé aux lésions postérieures ou malléolaires. Réalisation d'un parage du site receveur (Figure 1). Le premier temps consiste à repérer l'axe vasculo-nerveux du lambeau par une incision longitudinale prudente, la veine petite saphène est la plus facile à repérer, la situation du nerf étant variable en fonction de la hauteur du prélèvement. Le nerf et son plexus vasculaire sont liés et sectionnés séparément. La palette fascio-cutanée est alors relevée de proximal en distal, l'axe vasculo-nerveux, visible par transparence, guide la dissection (Figure 2).

**Figure 3:**
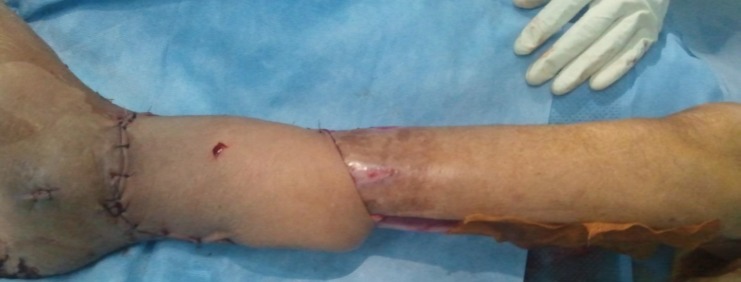
la mise en place du lambeau sur la perte de substance cutanée

**Figure 4:**
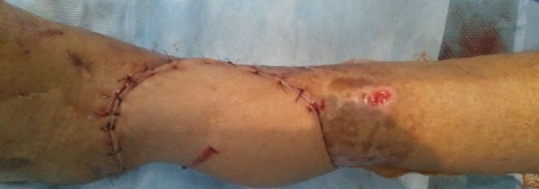
sevrage du lambeau après 3 semaines

**Figure 5:**
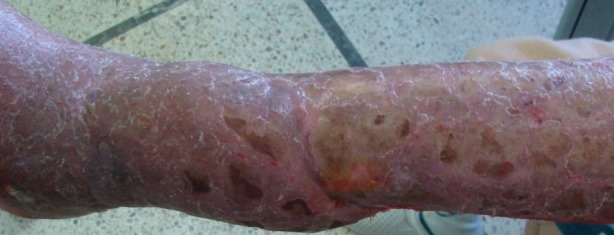
résultat après 2 ans

**Figure 6:**
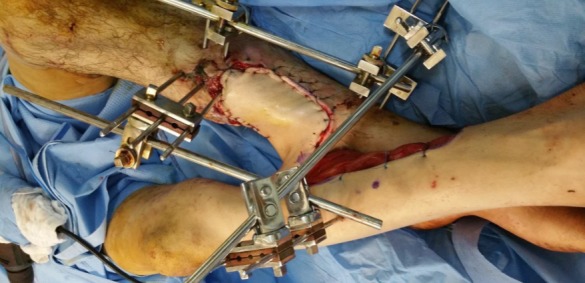
la couverture de la perte de substance cutanée par un lambeau fascio-cutané hétèro-jambier de type cross-leg

Pour atteindre l'extrémité distale de la jambe, le pédicule est retourné sur lui-même de 180°. La palette fascio-cutanée est posée sur le site receveur, sur lequel elle s'applique parfaitement grâce à la programmation préalable. Les sutures en tension ne sont pas acceptables, car source d'ischémie et de nécrose. L'affrontement des berges se fait derme à derme, les points sont simplement posés sans tension excessive (Figure 3). Exemple d'un cas de notre série ou le sevrage du lambeau fut réalisé 3 semaines après l'intervention (Figure 4). Le résultat final après 2 ans était satisfaisant (Figure 5). Les lambeaux fascio-cutanés hétèro jambiers type cross leg sont des lambeaux permettant de bénéficier d'un rapport longueur sur largeur très intéressant, facilitant ainsi beaucoup la mise en place sur le site receveur. Ils peuvent être levés avec un pédicule proximal ou distal. La technique du prélèvement du lambeau suscité classée également grade I selon Oberlin. Le patient est installé en décubitus dorsal, genou fléchi; la face médiale de jambe est exposée. Un garrot est installé à la racine de la cuisse. La levée du lambeau débute généralement par son bord antérieur, elle est menée de distal en proximal. L'incision est franche, de la peau au fascia inclus. Distalement, le nerf saphène et la veine grande saphène sont repérés, ligaturés et sectionné. Les artères musculo-cutanées et celles provenant de l'artère tibiale postérieure sont coagulées. La dissection se prolonge en proximal jusqu'à obtenir l'arc de rotation suffisant. Exemple de notre serie montrant une perte de substance du tiers inferieur de la jambe couverte par un lambeau fascio cutané hetero jambier type cross leg (Figure 6).

Lambeau hémi-soléaire médial a pédicule distal: est le muscle de référence dans les pertes de substance du tiers inferieur de la jambe. Le soléaire étant volumineux, seul l'hémi-soléaire médial, ou parfois l'hémi-soléaire latéral, est utilisé. Ce lambeau musculaire autorise la couverture des petites pertes de substance jusqu'au quart distal de la jambe, les régions péri-malléolaire médiale et rétro-achilléenne. La difficulté de réalisation de ce lambeau exige une sélection de patients jeunes avec de bons axes vasculaires et sans écrasement des masses postérieures. Il est néanmoins difficile et sa fiabilité n'est pas aussi certaine que celle de son homologue à pédicule proximal.

## Discussion

Le lambeau a pour but de fermer une perte de substance cutanée ou de reconstruire une structure amputée. Beaucoup de lambeaux sont également indiqués pour raccourcir le délai de traitement ou pour des raisons esthétiques [3]. Nous avons mené une étude comparative des différentes techniques de couverture utilisées dans la littérature et dans notre série, et ce selon plusieurs critères dont: l'âge, le sexe, les tares, le mécanisme et la dimension du défect.

**L'âge**: la moyenne d'âge, dans les différentes séries, varie entre 35 et 45 ans (Tableau 1).

**Tableau 1:** répartition des cas selon l’âge et le sexe

Auteurs	Sexe masculin	Sexe féminin	Moyenne d’âge (ans)
Vaienti [4]	80%	20%	20-35
Vaienti [5]	30%	70%	40-74
Voche [11]	66,66%	33,33%	Hommes: 52; Femmes: 77
Belmahi [9]	100%	0%	22-61
Penaud [7]	80%	20%	48
Sengezer [6]	100%	0%	20
Bous [10]	50%	50%	62-78
**Notre série**	**88,88%**	**11,11%**	**19-55**

**Le sexe**: dans notre série de patients comme dans plusieurs articles [4-11] nous retrouvons une atteinte des deux sexes, quoiqu'il y ait une prédominance masculine (Tableau 1).

**Les tares**: le choix d'une technique chirurgicale précise était basé sur le terrain et sur les tares entre-autre (Tableau 2).

**Tableau 2:** fréquence des tares chez les patients des différentes séries

Auteurs	% des patients présentant des tares	Tares
Vaienti [5]	50%	Obésité, Diabète
Voche [11]	80%	Diabète, HTA
Penaud [7]	14%	Diabète
**Notre Série**	**11,11%**	**Diabète**

**Mecanisme de la perte de substance cutanée**: comme il est constaté dans ces séries, la cause post traumatique est la plus retrouvée, que ce soit pour les traumatismes à haute énergie ou les accidents de la voie publique (Tableau 3).

**Tableau 3:** répartition des cas selon le mécanisme des pertes de substances cutanées

Auteurs	Post- traumatique	Ostéite	Autre (exérèse, brûlure …Etc.)
Vaienti [4]	+++		+
Vaienti [5]	+++	+	
Voche [11]	+++		+
Belmahi [8]	+++	+	
Penaud [7]	+++	+	+
Sengezer [6]	++++		
Bous [10]	++	++	
**Notre Série**	**++++**		**+**

**Topographie des pertes de substances cutanées**: dans notre série comme dans la littérature les localisations les plus fréquentes des pertes de substances sont la face antéro-interne et la face médiale du 1/3 inférieur de la jambe (Tableau 4).

**Tableau 4:** la topographie du défect cutané dans des séries étudiées

Auteurs	Topographie
Vaienti [5]	Face antéro-médiale+++ Face antéro-externe +
Voche [11]	Face antéro-médiale ++
Belmahi [8]	Face antéro-médiale+++ Face postérieure++
Penaud [7]	Face antéro-médiale ++ Face antéro-latérale +
Sengezer [6]	Face antéro-médiale ++
Bous [10]	Face antéro-médiale ++
**Notre série**	Face antéro-interne++ Face antéro-externe+ Face médiale+++ Face antérieure++

**Taille du défect**: la taille du défect est très variable dans les différentes séries étudiées. Elle dépend du mécanisme de la perte de substance, parage etc. Dans notre série la surface de la perte de substance cutanée est de 40 à 110 cm^2^ en moyenne. La série 14 a pris en charge les défects les plus petits (7-17 cm^2^), et ont soulevé l'intérêt de l'utilisation des petits lambeaux musculaires locaux ou des lambeaux fascio-cutanés. La série 16 par contre a pris en charge les défects les plus grands (150 cm^2^), et a choisi les lambeaux perforants en hélice (Tableau 5).

**Tableau 5:** les tailles moyennes du défect cutané dans des séries étudiées

Auteurs	Taille du défect en moyenne (cm^2^)
Vaienti [5]	5-32
Voche [11]	17
Belmahi [8]	7-10
Penaud [7]	81.3
Bous [10]	150
Notre Série	40-110

**Délai traumatisme/ couverture**: le délai de mise en place des lambeaux dans notre série est de 1 à 8 semaines. Ce délai retardé est rapporté dans d'autres séries en cas d'ostéite chronique [10, 12].

**Résultats et complications**: nous pouvons constater le succès des différentes techniques utilisées dans ces séries avec un taux de réussite variant entre 90% et 100%. Dans notre série un seul cas s'est compliqué d'une nécrose partielle avec persistance d'une perte de substance.

## Conclusion

Le tiers inférieur de la jambe reste une région anatomique où la chirurgie reconstructrice est particulièrement compliquée. Chaque temps du traitement est essentiel, en rappelant que le parage doit être fait précocement et doit être complet. Le choix du lambeau de couverture se fera en fonction du patient mais aussi de l'opérateur. Les résultats restent favorables pour la majorité des patients ayant bénéficié de différents types de lambeaux même en cas de grands défects.

### État des connaissances actuelles sur le sujet

- La couverture des pertes de substances cutanées du tiers inférieur de la jambe est compliquée, et ce dû à la pauvreté des tissus mous adjacents, la précarité de la vascularisation locale et l'exposition osseuse;
- Le choix d'une technique chirurgicale précise était basé sur le terrain et sur les tares entre-autre.

### Contribution de notre étude à la connaissance

- Contrairement au reste des séries de la littérature nous avons pu étudier plusieurs topographies de perte de substance avec une taille du defect importante;
- Nous avons un recul assez important.

## Conflits d’intérêts

Les auteurs ne déclarent aucun conflit d'intérêts.
